# The prognostic role of lymph node ratio in breast cancer patients received neoadjuvant chemotherapy: A dose-response meta-analysis

**DOI:** 10.3389/fsurg.2022.971030

**Published:** 2022-10-26

**Authors:** Jinzhao Liu, Yifei Li, Weifang Zhang, Chenhui Yang, Chao Yang, Liang Chen, Mingjian Ding, Liang Zhang, Xiaojun Liu, Guozhong Cui, Yunjiang Liu

**Affiliations:** ^1^ The Second Department of Thyroid and Breast Surgery, Cangzhou Central Hospital, Cangzhou, China; ^2^ Department of Pathology, Fourth Hospital of Hebei Medical University, Shijiazhuang, China; ^3^ Department of Breast Cancer Center, Fourth Hospital of Hebei Medical University, Shijiazhuang, China; ^4^ Hebei Provincial Key Laboratory of Tumor Microenvironment and Drug Resistance, Hebei Medical University, Shijiazhuang, China

**Keywords:** lymph node ratio, breast cancer, neoadjuvant chemotherapy, prognosis, meta-analysis

## Abstract

**Background:**

As neoadjuvant chemotherapy is widely used in breast cancer patients, the lymph node ratio has not been fully validated as a prognostic indicator of breast cancer received neoadjuvant chemotherapy. This study was conducted to investigate the prognostic value of lymph node ratio in breast cancer patients received neoadjuvant chemotherapy.

**Methods:**

Systematic searches were performed in the PubMed, Embase, and Cochrane Library databases until 15 December 2021 for studies on the association between lymph node ratio and the prognosis of breast cancer after neoadjuvant chemotherapy. Overall survival and disease-free survival were used as outcome events, and hazard ratio was chosen as the parameter to evaluate the correlation. The dose-response relationship was assessed by restricted cubic splines. In the subgroup analyses, which were used to explore potential heterogeneity among the included studies according to study region and sample size. Sensitivity analysis was performed to assess the stability of individual studies, and publication bias was determined with funnel plots, Begg’s test, and Egger&apos;s test. All statistical analyses were performed using Stata 15.1.

**Results:**

A total of 12 studies with 4,864 patients were included in this meta-analysis. In this study, high lymph node ratio was significantly associated with decreased overall survival (HR: 4.74; 95%CI: 3.36–6.67; *P *< 0.001) and disease-free survival (HR: 4.77; 95%CI: 3.69–6.17; *P *< 0.001). Moreover, the dose-response meta-analysis showed a linear association between higher lymph node ratio and shorter overall survival and disease-free survival in breast cancer patients after neoadjuvant chemotherapy.

**Conclusions:**

The meta-analysis suggested that high lymph node ratio was significantly associated with short overall survival and disease-free survival in breast cancer patients after neoadjuvant chemotherapy. Therefore, lymph node ratio is an independent predictive factor for the prognosis of breast cancer patients after neoadjuvant chemotherapy, which may better refine the cancer staging system.

## Introduction

Today, neoadjuvant chemotherapy is a standard treatment option for patients with locally advanced operable breast cancer and is increasingly used in early breast cancer (1). Neoadjuvant chemotherapy can not only convert inoperable disease to operable disease and reduce the scope of operable surgeries, but also provide confirmation of drug-sensitive disease, thereby guiding subsequent treatment with a view to improving patient outcomes (2). Although neoadjuvant chemotherapy improves overall survival (OS) and disease-free survival (DFS) in breast cancer patients, the prognosis of patients with lymph node positive breast cancer after neoadjuvant chemotherapy remain poor (3). The number of metastatic axillary lymph nodes is an important predictor of prognosis in patients with breast cancer, and accurate lymph node staging can provide an important reference value for guiding adjuvant therapy in patients. In clinical practice, due to the varying effects of neoadjuvant chemotherapy on axillary lymph node status and the technique of axillary lymph node dissection among clinicians, the number of axillary lymph nodes detected in postoperative patients after neoadjuvant chemotherapy is significantly lower than that in patients who did not receive neoadjuvant chemotherapy (4). According to the American Joint Committee on cancer (AJCC) staging of breast cancer, the recommendation regarding dissection of at least 10 lymph nodes after axillary lymph node dissection is clearly influenced by neoadjuvant chemotherapy. AJCC staging tends to underestimate the true status of axillary lymph nodes in breast cancer patients received neoadjuvant chemotherapy, thus affecting the accuracy of guiding treatment and assessing prognosis. Therefore, optimization of methods for assessing axillary lymph node status in breast cancer patients received neoadjuvant chemotherapy is essential.

Lymph nodes ratio (LNR) is defined as the ratio between positive lymph nodes and the total number of retrieved lymph nodes. It not only contains information about lymph node metastasis, but also has the degree of lymph node dissection. Previous studies have reported the independent prognostic value of the LNR in lung cancer, gastric cancer and colorectal cancer patients after neoadjuvant chemotherapy (5–7). Liu D et al. (8) proved that LNR is a prognostic factor for breast cancer in a meta-analysis, but the study did not conduct a subgroup analysis of patients received neoadjuvant chemotherapy, and the accuracy of LNR in evaluating the prognosis of patients received neoadjuvant chemotherapy is not clear. Some studies showed that LNR has important value in predicting the prognosis of breast cancer patients receiving neoadjuvant chemotherapy and its prognostic value was greater than that of current N staging (9, 10). However, Saxena et al. (11) found that LNR was an independent prognostic factor of breast cancer after neoadjuvant chemotherapy, and its prognostic value was poorer than that of ypN stage. Kim et al. (12) even denied the prognostic value of LNR in patients received neoadjuvant chemotherapy. The prognostic value of LNR in patients with breast cancer after neoadjuvant chemotherapy has been still controversial. Therefore, this study conducted a meta-analysis on the prognostic role of LNR in breast cancer patients received neoadjuvant chemotherapy for the first time, providing more comprehensive evidence for the prognostic value of LNR. We also performed a dose-response meta-analysis to examine the potential online relationship between LNR levels and prognosis after neoadjuvant chemotherapy.

## Materials and methods

### Search strategy

A comprehensive literature search of the PubMed, Embase and Cochrane Library databases was conducted to find relevant published articles about LNR prognostic prediction of breast cancer after neoadjuvant chemotherapy (updated to December 15, 2021). The retrieval strategy combines terms related to “breast cancer”, “neoadjuvant therapy”, “lymph node ratio” and “prognosis”. Additional studies were identified by hand searching the references of original articles and review articles.

### Selection criteria

All retrieved articles were first screened by title and abstract and irrelevant studies were excluded. Then, all the retrieved studies were screened by two reviewers according to the inclusion criteria and exclusion criteria. A third author would be consulted and the decision would be reached through discussions when a disagreement was encountered.

Inclusion criteria: (1) study design: retrospective or prospective cohort study; (2) participants: breast cancer patients received neoadjuvant chemotherapy and lymph node dissection; (3) primary outcomes: OS or DFS; (4) survival outcome was further explored regarding hazard ratio (HR) with confidence interval (CI), HR with *P* value, Kaplan-Meier curves or the needed data for calculating HR and CI;

Exclusion criteria: (1) study design: case-control or cross-sectional study; (2) participants: breast cancer patients complicated with other tumors or distant metastasis; (3) study types: case reports, conference summaries, review articles and reviews; (4) the same patient population were overlapped among publications (the studies with the largest sample size were included).

### Data extraction and quality assessmenth

The following information were extracted from the included studies: first author, publication year, country, study design, sample size, follow-up time, tumor stage, cut-off value, HRs and 95% CIs for OS and/or DFS. The quality of the included studies in this meta-analysis was assessed according to the Newcastle-Ottawa Quality Assessment Scale (NOS). This scale evaluated each study in three domains including the selection of the participants, the comparability between the groups and the outcome of interest for cohort study. The NOS scores range from 0 to 9, and studies with NOS scores > 6 were considered high quality (13).

### Statistical analysis

In this meta-analysis, HR and its 95% CIs were used to evaluate the relationship between LNR level and prognosis in breast cancer patients after neoadjuvant chemotherapy. For each study, the HRs comparing the highest with the lowest category were then displayed in a forest plot. In addition, we performed a dose-response meta-analysis to assess whether LNR was associated with worse OS and DFS in breast cancer patients receiving neoadjuvant chemotherapy. When the included studies reported only the total number of cases and the number of cases in each category, the number of person years in each category was calculated using the method proposed by bekkering Ge et al. (14) and Aune D et al. (15). According to the LNR interval given in the included study, we designated the middle value of the upper and lower boundaries of each category as the average LNR level.

The Cochran’s *Q* test and *I*^2^ statistics were used to analyze heterogeneity between studies; *P* < 0.05 or *I*^2 ^> 50% suggested significant heterogeneity among the included studies. If significant heterogeneity existed, a random effect model was selected; otherwise, the fixed-effects model was used. The subgroup analysis was also conducted to explore the source of heterogeneity based on the study area (Chinese or non-Chinese) and sample size of studies (≤300 vs. >300). The possibility of publication bias was evaluated by visual screening of the Begg’s funnel plot, and both Begg’s test and Egger’s test were used to evaluate the publication bias. A significance of *P* < 0.05 indicated the possibility of publication bias (16, 17). To further evaluate the robustness of our results, we conducted sensitivity analysis. Sensitivity analysis was performed to explore the potential influence of each individual study on the overall results by deleting one single study each time from the pooled analysis.

Stata se 15.1 (Stata company, Texas College Station, USA) was used for statistical analysis. The study was reported according to the PRISMA Checklist (Stewart et al., 2015).

## Results

### Selection and characteristics of included studies

**Figure 1:**
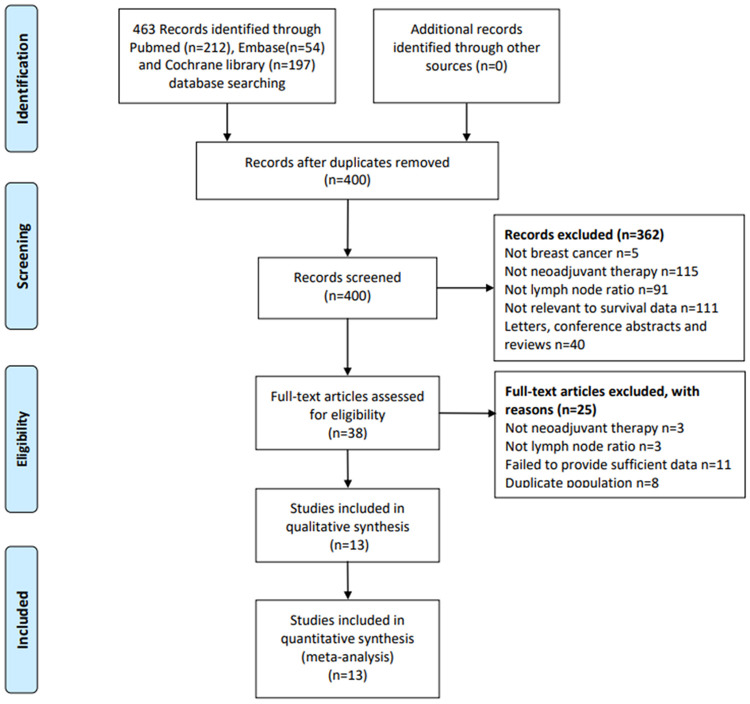
Flowchart of the study search and selection.

A total of 463 articles were retrieved on the initial literature search, of which 212 were retrieved from PubMed, 54 from Embase and 197 from the Cochrane Library. After the exclusion of duplicate studies and non-relevant studies based on a screening of article titles and abstracts, 38 potentially relevant studies were retrieved for full review. According to the pre-established inclusion and exclusion criteria, 13 studies, involving 4,864 breast cancer patients, were included in this study (9–11, 18–27). The flow diagram of the literature search was shown in Figure 1. These studies were published between 2009 and 2021. Only one study was a prospective study, and the rest were retrospective studies. Of the 12 studies, 10 studies reported OS and 11 reported DFS. With regard to the study area, four studies were conducted in Chinese (10, 19, 22, 27), while the remaining nine studies were conducted in non-Chinese countries. The median follow-up ranged from 24 to 87 months. The LNR thresholds used in the included studies ranged from 0.1 to 0.8, with most (10/12) using LNR thresholds of 0.2 and 0.65. Overall quality of the included studies was good, and NOS scales ranged from 6 to 8. Table 1 provides the basic characteristics of included studies.

**Table 1:** Characteristic of the included studies.

Study	Year	Country	Study design	Study period	Sample size	Tumour stage	Follow-up time	Cut-off value	Endpoint	Quality scale
Keam	2009	Korea	Prospective	2002–2007	205	Stage II/III	Median 28.9 months	0.25	OS, DFS	6
Saxena	2011	Geneva Kuala, Singapore, Malaysia	Retrospective	1990–2007	314	Stage I/II/III	NA	0.2, 0.65	OS	7
Chen	2014	China	Retrospective	1999–2009	569	Stage II/III	Median 48 months	0.2, 0.4, 0.8	DFS	7
Tsai	2016	America	Retrospective	2003–2014	428	NA	Mean 36.9 months	0.2, 0.65	DFS	7
Cho	2018	Korea	Retrospective	2006–2015	236	Stage I/II/III	Mean 54 months	0.2, 0.65	OS, DFS	7
Agarwal	2019	India	Retrospective	2004–2014	224	Stage II/III	Median 61 months	0.2, 0.65	OS, DFS	8
Lai	2019	China	Retrospective	2009–2012	339	Stage II/III	Median 62.3 months	0.4, 0.8	DFS	7
Soran	2019	America	Retrospective	2009–2014	179	Stage I/II/III	Median 24 months	0.2	OS	7
Tonellotto	2019	Brazil	Retrospective	2008–2009	628	Stage II/III	Median 58 months	0.2, 0.65	OS, DFS	7
Ai	2020	China	Retrospective	2007–2014	306	Stage II/III	Median 78 months	0.2, 0.65	OS, DFS	7
Gabriel A	2020	Peru	Retrospective	2000–2014	171	Stage II/III	Median 87 months	0.2, 0.65	OS, DFS	6
Silva	2021	Brazil	Retrospective	2010–2014	171	Stage II/III	Median 62.5 months	0.2, 0.65	OS, DFS	7
Li	2021	China	Retrospective	2008–2018	282	Stage I/II/III	Mean 63 months	0.2, 0.65	OS, DFS	7

### Relationship between LNR and prognosis of breast cancer patients received neoadjuvant chemotherapy

**Figure 2:**
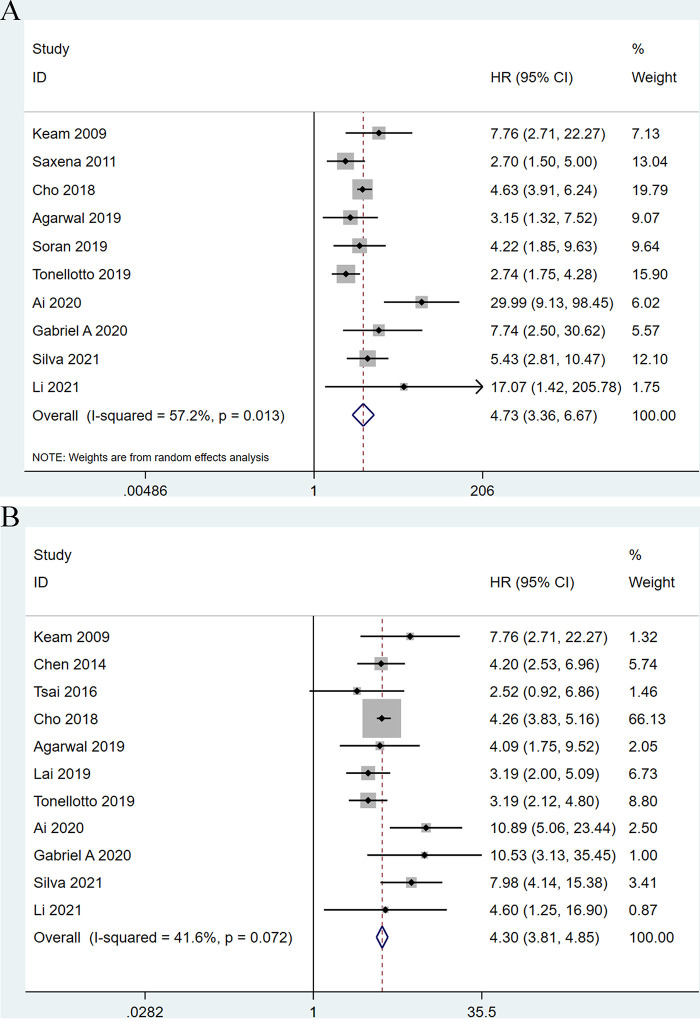
Forest plots show the association between lymph node ratio and overall survival (A), disease-free survival (B).

Among the 13 eligible studies, 10 studies (9–11, 18, 21, 23–27) explored the association between LNR and OS outcomes. Meta-analysis has demonstrated that a significant correlation between higher LNR and shorter OS of breast cancer patients received neoadjuvant chemotherapy (HR: 4.74; 95%CI: 3.36–6.67; *P *< 0.001) with significant heterogeneity (*I*^2 ^= 57.2%; *P *= 0.013) (Figure 2A).

Moreover, 11 studies (9, 10, 18–22, 24–27) examined the association between higher LNR and shorter OS of breast cancer patients after neoadjuvant chemotherapy. The results showed a significant association (HR: 4.77; 95%CI: 3.69–6.17; *P *< 0.001) with no heterogeneity (*I*^2 ^= 41.6%; *P *= 0.072) (Figure 2B).

### Subgroup analysis

**Figure 3:**
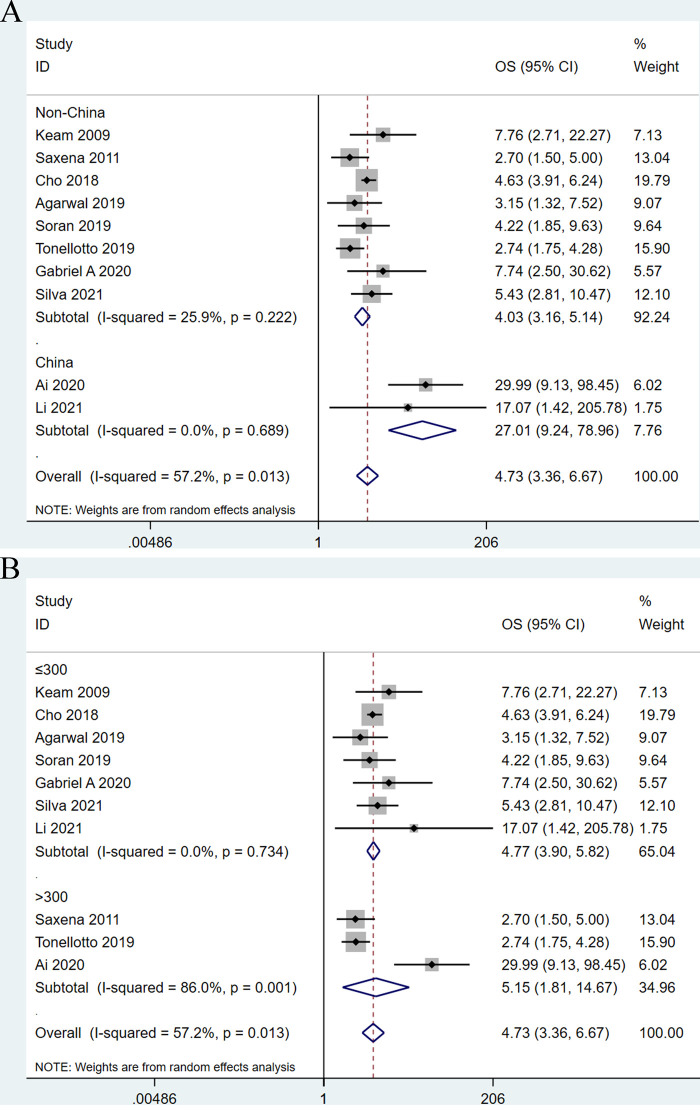
Forest plots show the association between lymph node ratio and overall survival stratified by the studied area (A) and sample size (B).

In order to explore the potential sources of heterogeneity of the combined HR for OS, we conducted subgroup analyses through stratifying eligible studies by study area (Chinese vs. non- Chinese) and sample size (≤300 vs. >300). When divided into two subgroups by study area, the heterogeneity between studies disappeared. With regard to nation, higher LNR was significantly correlated with shorter OS (HR: 27.01; 95%CI: 3.36–6.67; *I*^2 ^= 0.0%; *P *< 0.001) in Chinese patients compared with non-Chinese patients (HR: 4.03; 95% CI: 3.16–5.14; *I*^2 ^= 25.9%; *P *< 0.001) (Figure 3A). Based on the subgroup analysis by sample size, this subgroup analysis did not alter the prognostic role of LNR in OS substantially, but significant heterogeneity remained across studies, as shown in Figure 3B.

### Dose-response analysis

**Figure 4:**
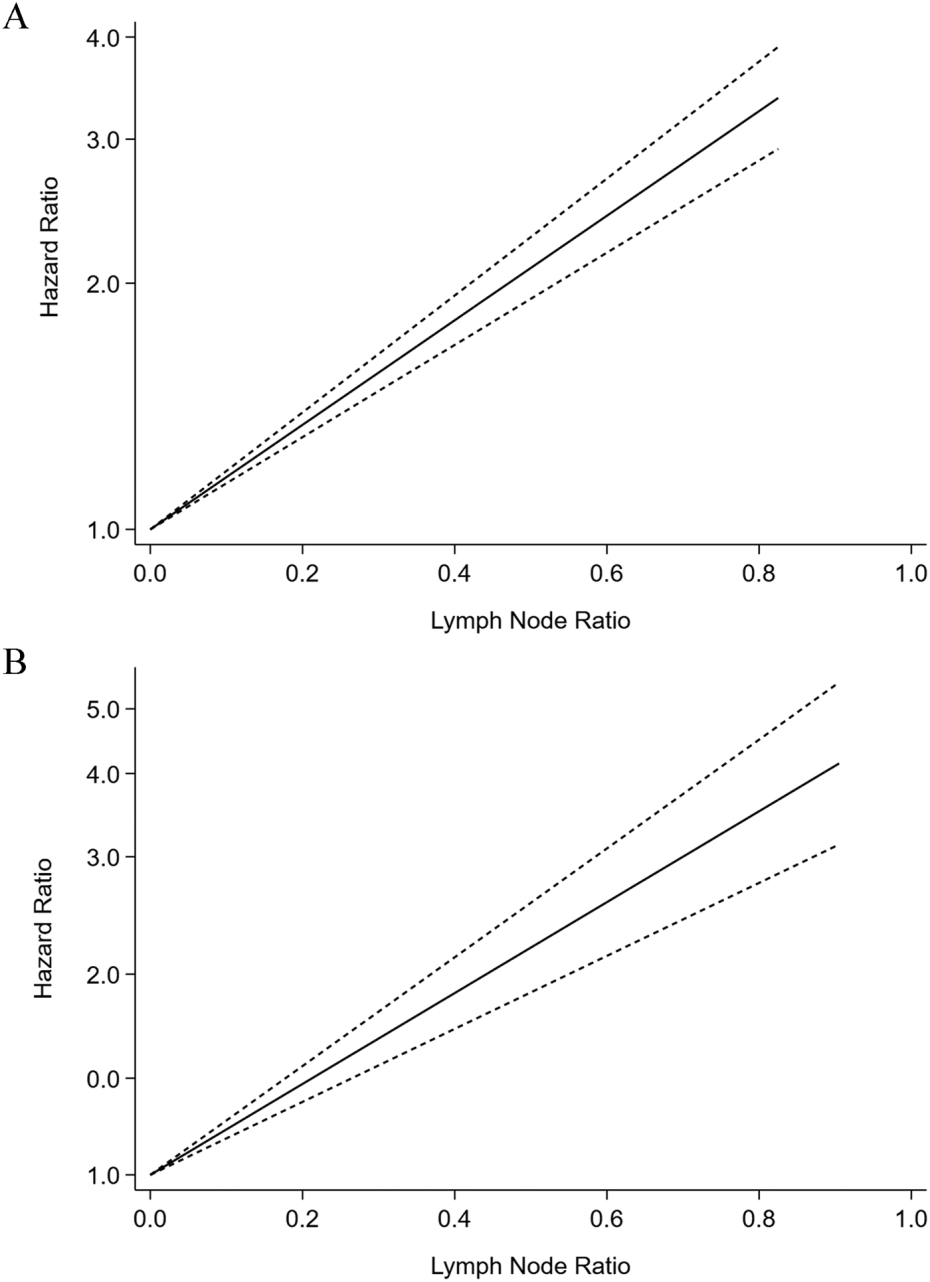
Dose-response meta-analysis of the prognostic role of lymph node ratio in overall survival (A) and disease-free survival (B) of the breast cancer patients after neoadjuvant chemotherapy.

Six studies were considered ineligible for inclusion in the dose-response analysis due to a lack of information regarding prognosis of participants or provided LNR levels for less than three categories. Therefore, six cohort studies were eligible to had required data for dose-response analysis. We found a significant linear relationship between higher LNR levels and shorter OS after neoadjuvant chemotherapy (HR = 1.47, 95%CI:1.298–1.646, *P *< 0.001), and there was no evidence of heterogeneity in the study (*Q* = 2.17, *P *= 0.83) (Figure 4A). A total of nine studies participated in the dose-response analysis of the relationship between LNR level and DFS after neoadjuvant chemotherapy. The results showed that there was a linear relationship between higher LNR level and shorter DFS (HR = 1.50, 95% CI: 1.394–1.616, *P *< 0.001), and the heterogeneity across the studies was significant (*Q* = 20.72, *P *= 0.008) (Figure 4B).

### Sensitivity analysis and publication bias

**Figure 5:**
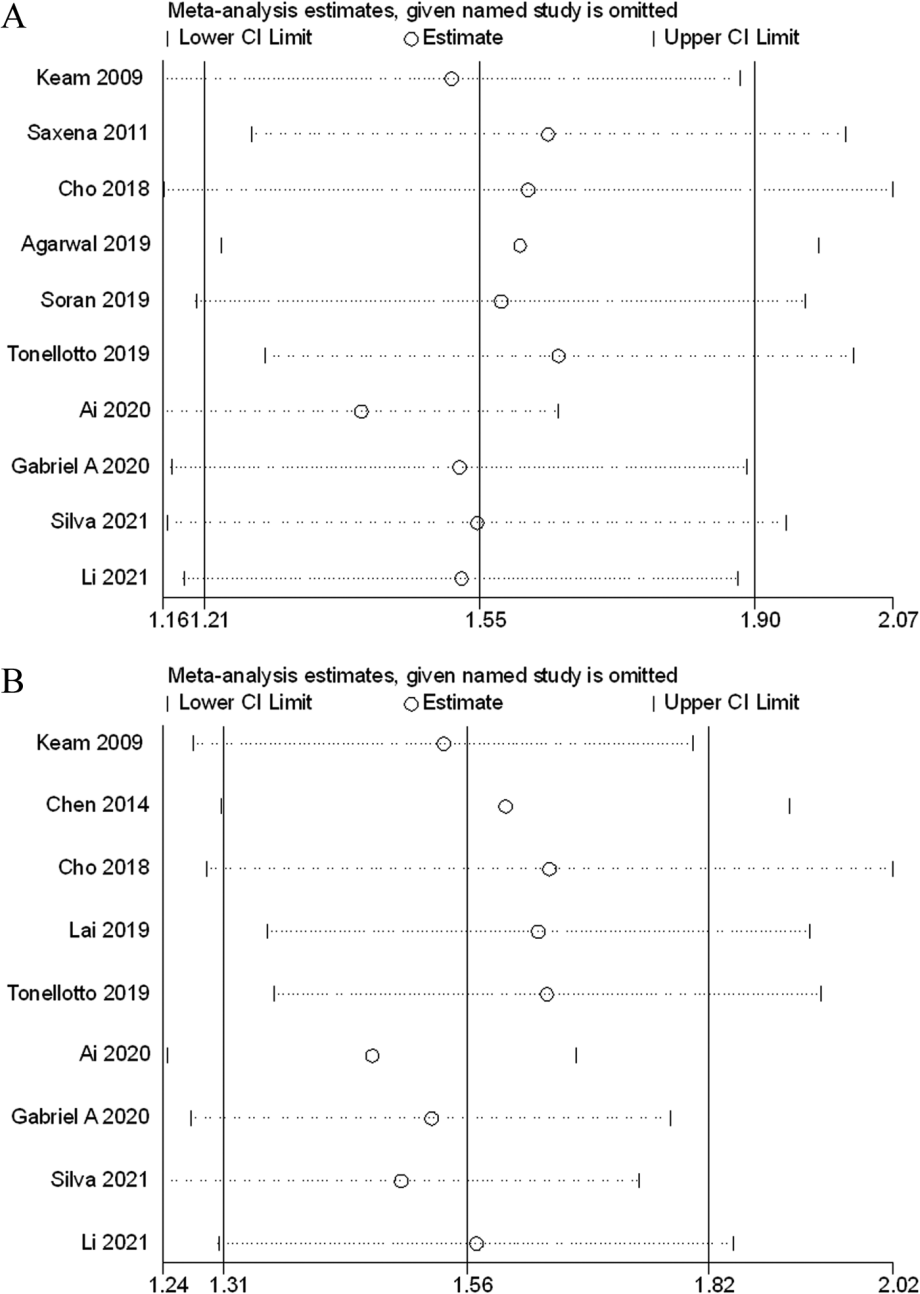
Sensitivity analysis of the association between lymph node ratio with overall survival (A), disease-free survival (B).

**Figure 6:**
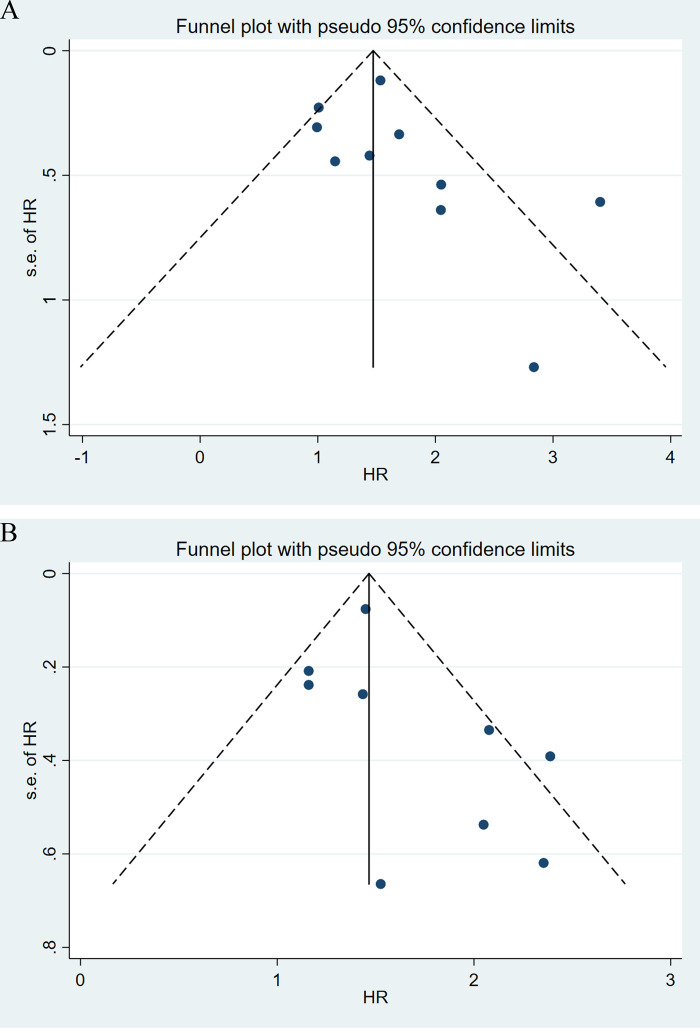
Funnel plot of the association between lymph node ratio with overall survival (A), disease-free survival (B).

Sensitivity analyses were performed next. A single study involved in the meta-analysis was deleted each time to unveil the influence of the individual data set to the pooled HRs. In the current study, removing any of the included studies had no significant impact on the meta-analytic results, indicating the robustness of the results (Figure 5). The Begg’s funnel showed no significant asymmetry for all included studies (Figure 6). Similarly, the quantitative evaluation results of Begg’s test and egger’s test showed that there was no statistically significant publication bias in the studies reporting OS (egger’s test: *P *= 0.479; Begg’s test: *P *= 0.858) and DFS (egger’s test: *P *= 0.194; Begg’s test: *P *= 0.118).

## Discussion

Today, neoadjuvant chemotherapy treatment is widely accepted as a standard treatment for locally advanced breast cancer and plays an important role in the comprehensive treatment of breast cancer (28). The management of the axilla after neoadjuvant chemotherapy is still dominated by axillary lymph node dissection, which aimed to establish nodal status and guide adjuvant treatment indication to maximize survival and regional control of cancer in breast cancer patients (29). The National Comprehensive Cancer Network (NCCN) defines an adequate axillary lymph node dissection as retrieval of least 10 lymph nodes to accurately stage the axilla (30). Rosenberger LH et al. (31) found that fewer dissected lymph nodes were associated with poorer OS in breast cancer patients with positive axillary lymph nodes, possibly due to insufficient axillary staging and missed opportunities for adjuvant therapy. Previous studies reported the lower lymph node yield after axillary lymph node dissection in breast cancer patients received neoadjuvant chemotherapy, and found that neoadjuvant chemotherapy was an important factor associated with dissection of fewer than 10 lymph nodes (32, 33). Neoadjuvant chemotherapy can induce histomorphological changes within lymph nodes regarding the features lymphoid depletion, diffuse fibrosis, disruption or blockage of lymphatic vessels, calcifications and signs of bleeding (34, 35). These histomorphological changes may lead to decreased lymph node harvest rates. Erbes t et al. found that lymphoid cell depletion was an important factor with the low lymph node yield after neoadjuvant chemotherapy (33). This might be explained by the fact that lymphoid cell depletion will lead to shrinkage of lymph nodes as well as to regression of lymphoid tissue. In a retrospective study using data from the National Cancer Database, it was found that the yield of axillary lymph node dissection was significantly lower in patients received neoadjuvant chemotherapy than those who underwent surgery alone, and that patients received neoadjuvant chemotherapy were more likely to not meet the criteria of axillary lymph node dissection. In addition, the study also found that low lymph node yield was independently associated with pCR of the primary tumor (36). With the development of chemotherapy regimens and targeted anti-HER2 treatment, the primary tumor and axillary pCR rates have increased substantially. A previous study found that fewer than 10 lymph nodes were found in 41.7% of 139 breast cancer patients who underwent axillary dissection and received neoadjuvant pertuzumab, however in patients who received neoadjuvant chemotherapy but did not receive pertuzumab, only 18.6% of patients had less than 10 axillary lymph nodes dissected (37). Therefore, the low lymph node yield will underestimate the number of metastatic lymph nodes and may lead to improper prediction of prognosis and improper treatment. In the era of neoadjuvant treatment, the 10-lymph node guideline for axillary lymph node dissection in breast cancer may need to be revised.

Currently, lymph node status remains an important factor in the AJCC prognostic staging and remains an essential determinant of adjuvant treatment decision-making (38). Lymph node staging is still based on positive lymph node count in breast cancer patients received neoadjuvant chemotherapy, but the varying effects of neoadjuvant chemotherapy on axillary lymph nodes is not considered. Therefore, LNR can overcome the limitation of only taking positive lymph node count, improve and complement the assessment of ypN stage in post-neoadjuvant chemotherapy breast cancer patients, especially for those with fewer than 10 lymph nodes dissected. To the best of our knowledge, this is the first meta-analysis to demonstrate the prognostic role of LNR in neoadjuvant chemotherapy for breast cancer patients. The results of the present study prove that increased LNR levels can predict the shortening of OS and DFS in breast cancer patients after neoadjuvant chemotherapy. However, we found that there was heterogeneity between studies explored the relationship of LNR and OS for patients received neoadjuvant chemotherapy. Subgroup analysis of study area and sample size also demonstrated that high LNR level was associated with worse OS, and the heterogeneity disappeared when divided by area. Among them, the correlation between high LNR level and worse OS was greater in Chinese population than in non-Chinese population after neoadjuvant chemotherapy. The sensitivity analysis confirmed the reliability and stability of the meta-analysis. In addition, LNR level showed a linear correlation with shorter OS and DFS after neoadjuvant chemotherapy. Our findings demonstrated the importance of LNR in the prognosis of breast cancer after neoadjuvant chemotherapy. Therefore, we suggest that LNR should be included as a prognostic parameter in future staging systems for breast cancer after neoadjuvant chemotherapy.

Although many studies mainly explore the relationship between LNR and the prognosis of breast cancer after neoadjuvant chemotherapy, the existing evidence of reliable and reproducible LNR cut-off values is inconsistent. The prognostic value of LNRs was calculated for values ranging from 0.05 to 0.95 by Cox regression analysis and validated by bootstrapping. Vinh hung V et al. calculated the prognostic value of LNRs for values ranging from 0.05 to 0.95 by Cox regression analysis and recorded the difference in likelihood between the critical value model and AIC model, and identified a pair of critical values associated with the least negative difference in likelihood (0.20 and 0.65) (39). Kim JY et al. found that 0.25 and 0.55 as the most significant cut-off values of LNR associated with prognosis, by minimum *P*-value approach (40). According to X-tile software results, Xiao XS et al. found that the optimal cut-off values for LNR were 0.3 and 0.8 (41). Until now, the different cut- off for LNR among studies due to different statistical methods for optimal cut-off for LNR. Previous investigations of the prognostic value of LNR in breast cancer have focused on patients who did not receive neoadjuvant chemotherapy, while few studies of patients who received neoadjuvant chemotherapy have been conducted. The cut-off values for LNR of the included studies in this meta-analysis mostly were 0.20 and 0.65, but no study evaluated whether they could well predict the prognosis of breast cancer patients after neoadjuvant chemotherapy. Regarding the selection of the optimal cut-off value for predicting the prognosis of breast cancer patients, more large samples and high-quality studies in the future are needed to stratify and evaluate the effect of different LNRs on the prognosis of breast cancer patients after neoadjuvant therapy, and to determine the optimal LNR cut-off value for clinical practice.

The present meta-analysis has certain limitations. First, most of the studies included in our meta-analysis were retrospective. The different results of these studies may be caused by population heterogeneity, different neoadjuvant chemotherapy regimens and cycles, different number of axillary lymph node resections and varying surgical and pathological quality across medical centers. Second, the cut-off value for defining LNR in each included study is quite different, which may have contributed to heterogeneity. Third, the value of LNR after neoadjuvant chemotherapy is vague due to treatment impact and the change of lymph node metastases. Therefore, more prospective studies with better designed trials would be warranted for future LNR studies. Finally, due to insufficient information in the included studies, this study could not analyze the relationship between LNR levels and breast cancer prognosis based on a comprehensive analysis including histological grade, molecular typing, TNM stage, or adjuvant therapy.

In conclusion, the results of the present meta-analysis suggest that the level of LNR is a predictive factor for response in breast cancer patients received neoadjuvant chemotherapy. LNR can be used as a supplement to TNM staging in breast cancer patients after neoadjuvant chemotherapy and improve the accuracy of tumor staging.

## Data Availability

The original contributions presented in the study are included in the article/Supplementary Material, further inquiries can be directed to the corresponding author/s.
